# Investigating the potential of the isometric handgrip exercise as a test of cardiovascular reactivity to blood pressure fluctuations

**DOI:** 10.1186/s12871-026-03880-2

**Published:** 2026-05-01

**Authors:** Anna C. Zimmermann, Dominik P. Guensch, Julia Abegg, Louis Setz, Bernd Jung, Mario D. Neuenschwander, Christoph D. Utz, Leonard Grob, Hendrik von Tengg-Kobligk, Adrian T. Huber, Kady Fischer

**Affiliations:** 1 Department of Anaesthesiology and Pain Medicine, Inselspital, Bern University Hospital, University of Bern, Freiburgstrasse 10, Bern, 3010 Switzerland; 2 Department of Diagnostic, Interventional and Paediatric Radiology, Inselspital, Bern University Hospital, University of Bern, Bern, Switzerland; 3 Translational Imaging Center (TIC), Swiss Institute for Translational and Entrepreneurial Medicine, Sitem-Insel, Bern, Switzerland; 4 Department of Radiology and Nuclear Medicine, Lucerne Cantonal Hospital, University of Lucerne, Lucerne, Switzerland

**Keywords:** Cardiovascular magnetic resonance, Blood pressure, Isometric, Handgrip exercise, Aortic function, Ventricular function, Atrial function

## Abstract

**Background:**

In the perioperative setting, blood-pressure fluctuations can cause adverse outcomes, underscoring the need to identify vulnerable patients preoperatively. Imaging-based cardiovascular diagnostic tests are rapidly evolving, yet few are developed with anaesthesia in mind. This highlights an opportunity to develop techniques that evaluate the cardiovascular response to changes in blood pressure, but early feasibility studies in healthy controls are needed before clinical application. In combination with cardiovascular magnetic resonance (CMR) imaging, the isometric handgrip test has the potential to comprehensively assess how the heart and ascending aorta respond to transiently elevated blood pressure. We investigated the effect of an isometric handgrip exercise on myocardial tissue features with atrial, ventricular, and aortic function and coupling in healthy adults, assessed by CMR.

**Methods:**

In a single-centre observational study, 45 healthy adults underwent a CMR exam. Following acquisition of non-invasive blood pressure as well as cardiac and aortic rest images, participants sustained a grip of a dynamometer at 30% of their maximal strength. After a 2.5 min adjustment period, identical images were acquired. Myocardial blood volume and oxygenation were measured using parametric mapping and oxygenation-sensitive imaging. Furthermore, aortic function was assessed via vascular strain and flow parameters, while atrial and ventricular function were assessed through long-axis shortening. Coupling was calculated from atrial, ventricular and aortic parameters.

**Results:**

The isometric handgrip exercise improved left ventricular and left atrial systolic (-18.4±2.6% to -19.3±3.3%, *p* < 0.05 and 36.2±6.4% to 39.6±7.9%, *p* < 0.01) and late diastolic function (4.4 ± 1.7% to 5.1 ± 1.6%, *p* = 0.04 and −11.5 ± 4.5% to -14.8 ± 5.3%, *p* < 0.01), assessed by long-axis shortening. Aortic strain, reflecting vascular elasticity, decreased (31.0±11.3% to 26.1±12.3%, *p* < 0.01). The ventricular-arterial systolic coupling index dropped (31.0 ± 11.3% to 26.1 ± 12.3%, *p* < 0.01), while atrio-ventricular coupling remained unchanged. While myocardial blood volume did not change (T1: 1193±25ms to 1194±22ms, *p* = 0.77), myocardial oxygenation decreased (-2.9% [95%CI: -4.4, -1.3], *p* = 0.02).

**Conclusions:**

The isometric handgrip exercise effectively increased afterload and elicited augmented cardiovascular function and myocardial oxygen demand in healthy adults. Our data demonstrate the physiological changes expected from increased afterload in healthy hearts. Future studies in patient cohorts are warranted to investigate if it has potential as diagnostic tool for preoperative stress testing.

**Supplementary Information:**

The online version contains supplementary material available at 10.1186/s12871-026-03880-2.

## Introduction

Imaging-based cardiovascular diagnostic tests are rapidly evolving, yet few are developed with anaesthesia in mind. This highlights an opportunity to develop techniques for preoperative risk assessment, yet these require early feasibility studies in healthy controls before clinical application. With the increased prevalence of cardiovascular risk factors such as obesity, sedentary lifestyle, or disturbed glucose metabolism, more patients presenting for non-cardiac surgery may be susceptible to adverse cardiovascular outcomes during surgery [1]. Subclinical cardiovascular dysfunction can be unmasked through haemodynamic fluctuations [2]. The perioperative environment contains many haemodynamic stressors that lead to blood pressure fluctuations, both through anaesthesia and surgery, including intubation, pharmacological agents and blood loss. Not only perioperative hypotension is associated with adverse outcomes, but perioperative hypertension and blood pressure variability are as well [3]. Perioperative hypertension is not a rare occurrence as findings from a survey of 339 anaesthesiologists indicated that more than half answered they encounter perioperative hypertension in 20–50% of their surgeries [4]. Fluctuations in blood pressure can influence cardiac function through several mechanisms, including changes in preload, afterload, and perfusion pressure [5]. Patients with impaired cardiovascular function are more likely to have a reduced functional reserve impeding proper adjustment to the higher perfusion demand therefore predisposing patients to perioperative complications including haemodynamic instability, ischaemia, new-onset heart failure, and ultimately unfavourable outcomes [3, 6]. Therefore, it is vital for the anaesthetist to understand the patients’ cardiac and vascular health and reactivity to blood pressure changes prior to surgery in order to anticipate and counteract adverse cardiovascular reactions.

To identify patients with cardiovascular dysfunction preoperatively, there are currently a variety of stress tests in use; however, the literature is mixed on the effectiveness of preoperative stress testing [7]. This can be due to the heterogeneity of stressors applied and the modality used to quantify the cardiovascular response. The use of cardiovascular magnetic resonance (CMR) for diagnostic imaging is rapidly increasing worldwide [8]. Unlike modalities such as electrocardiograms and even echocardiography, CMR allows for a comprehensive assessment of cardiovascular structure, tissue features such as oxygenation and oedema as well as whole-heart function [9, 10]. Especially when applying parametric imaging sequences to quantify myocardial tissue, it has a clear benefit in identifying diffuse and microvascular changes on a millimeter scale [11, 12]. Furthermore, structures adjacent to the heart, like the aorta, can be assessed in the same exam. Due to rapid imaging techniques, changes in myocardial tissue features and cardiovascular function can be assessed following a stimulus or stress. Assessing not only individual chambers but coupling across the heart can give further insight into the efficiency of cardiovascular function.

Typically, diagnostic exams use injected pharmacological vasodilators, which are designed to assess myocardial perfusion, but may not adequately represent the blood pressure changes experienced perioperatively. Standard exercise testing, like the ergometer or treadmill, induces rapid changes in blood pressure, but these are not ideal for CMR, as the physical constraints of the bore limit leg motion, and image acquisition relies on no movement of the chest. An isometric handgrip exercise test, which is known to increase blood pressure, presents an ideal solution [13–15]. It requires minimal equipment and is therefore inexpensive; it induces minimal movement and can be performed in the supine position. It can also be adjusted to the patients’ fitness level by using maximal grip strength as a reference and using a standardized fraction as target strength. Therefore, it has ideal characteristics to be used for preoperative haemodynamic testing. However, before any studies assessing its clinical applicability for the field of anaesthesia can be undertaken, preliminary investigations in a non-operating environment are needed in participants with healthy cardiovascular systems. It must first be determined whether the blood pressure changes elicited by the exercise produce measurable changes in function and oxygenation across the heart.

Accordingly, this study aimed to investigate the feasibility and impact of a five-minute isometric handgrip exercise on myocardial, ventricular, atrial, and aortic function and tissue features assessed by CMR in healthy adults.

## Methods

### Study and participant details

Healthy volunteers (*n* = 45) were prospectively recruited from 08.2020 to 10.2021 into a single-centre observational study to undergo a CMR exam for research purposes. They had to be aged between 18 and 45 years and were excluded if they presented any contraindications for magnetic resonance imaging (MRI) according to current guidelines, knowledge or suspicion of pregnancy, or consumption of coffee within 12 h of imaging [10]. All participants provided informed written consent. This study was approved by the Ethics Committee for Research on Humans in the Canton of Bern, Switzerland and complies with the declaration of Helsinki. This analysis was conducted as part of a prospective observational study, from which the present outcome was defined a priori as a secondary endpoint of the main study.

### Imaging protocol

**Fig. 1:**
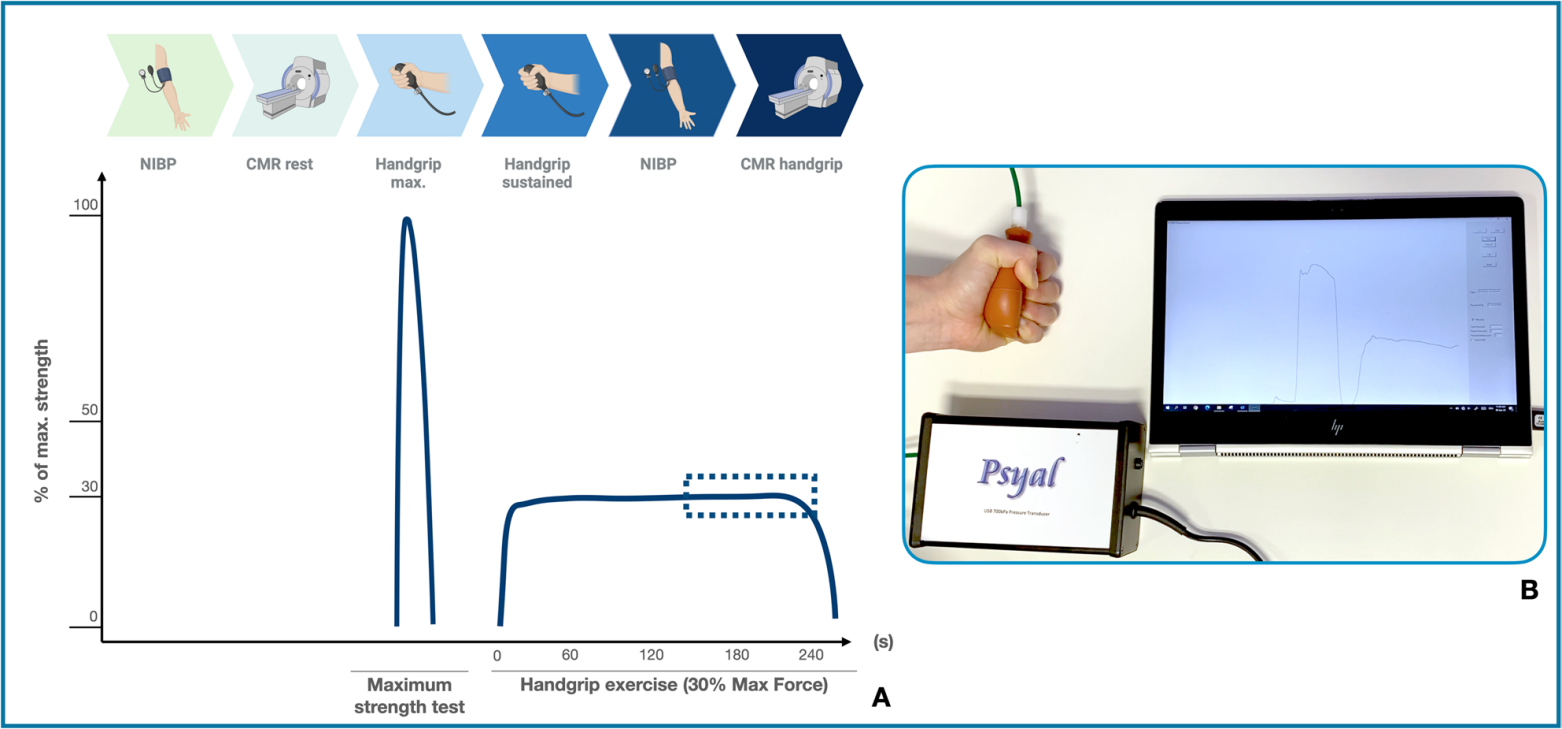
Handgrip Protocol. **A**: After the acquisition of non-invasive blood pressure (NIBP) and rest cardiovascular magnetic resonance images (CMR), the maximal handgrip strength was assessed. Participants were then instructed to maintain a handgrip at 30% of their individual maximum strength (**B**). After a 2.5-minute waiting period, blood pressure and handgrip images were reobtained

Participants underwent a single CMR visit (3.0 Tesla scanner, MAGNETOM Prisma, Siemens Healthineers, Germany). Patients laid in a supine position and after localization, images assessing cardiovascular function and myocardial tissue were obtained in a resting state (Fig. 1). Non-invasive blood pressure was obtained at the same time as the baseline measurement. For the isometric handgrip exercise, participants were first instructed to clutch the MRI-compatible dynamometer (Psyal Limited, www.psyal.co.uk, United Kingdom) in their dominant hand at maximum strength for 10 s while lying in the MRI machine. Next, the participants were instructed to maintain a squeeze of the dynamometer at 30% of their individual maximum capacity. Pressure readings from the dynamometer were simultaneously monitored in the control room by the research team, and if the applied pressure dropped or rose, participants were instructed through the loudspeaker system to adjust their grip force accordingly. A failure to perform the handgrip exercise was defined as either the participant indicating inability to complete the test or the force dropping below the target tolerance band of 25% of maximum force for more than 5 s (supplemental Fig. 1). After 2.5 min images identical to the rest imaging protocol were acquired. During image acquisition the participants maintained handgrip pressure, and blood-pressure was reobtained. CMR imaging parameters are provided in supplemental methods.

### CMR image analysis

**Fig. 2:**
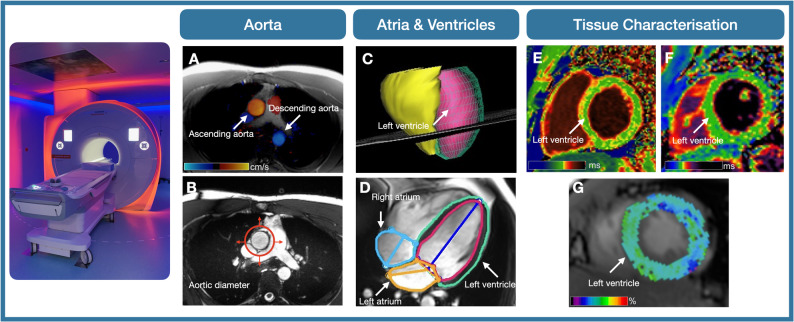
Multiparametric CMR Exam. The ascending aorta was assessed with a 2D flow (**A**) and cine image (**B**) perpendicular to the aorta. In addition to standard biventricular volumetry (**C**), a four-chamber analysis module (**D**) assessed long-axis shortening of both atria and the left ventricle, while right ventricular function was quantified with tricuspid annulus planar excursion indexed to right ventricular length. Finally, changes in tissue water content were assessed with T1(**E**) and T2 (**F**) mapping, while oxygenation sensitive assessed the myocardial oxygenation response (**G**)

Images were recoded and analysed by blinded readers and performed primarily using Circle Cardiovascular Imaging (version 5.17–6.2, Calgary, Canada, Fig. 2). Twelve images were randomly chosen and reanalysed by a second blinded reader.

#### Aortic function

2D blood flow, velocity and stroke volume through the ascending aorta was obtained from phase contrast images, without using background correction. Aortic cines from the same plane were analysed for aortic size, strain, and distensibility, and a derived value of vascular age was noninvasively calculated (CardioInspect/ArtFun+, Imageens, France) [16].

#### Ventricular and atrial function

Standard parameters of left (LV) and right ventricle (RV) function, including stroke volumes and ejection fraction, were acquired using real-time short-axis cines. From a standard segmented 4-chamber long-axis cine, long axis shortening (LAS) of the left (LA) and right atria (RA) and the LV were calculated throughout the cardiac cycle [17]. RV function was measured by tricuspid annular planar excursion normalized to the right ventricular length at end-diastole (RV-TAE_i_). For all four cardiac chambers, peak systolic as well as early and late diastolic function was identified [18]. Coupling indices were assessed by calculating the ratios between atrio-ventricular and ventriculo-atrial parameters.

#### Myocardial tissue analysis

Changes in parametric T1 and T2 mapping were quantified to assess changes in LV myocardial blood volume as these sequences are sensitive to tissue free water content. As a marker of relative changes in LV myocardial oxygenation, signal intensity from oxygenation-sensitive CMR images were reported as a percent-change between rest and stress [19, 20].

### Data and statistical analysis

Measurements between baseline and exercise were compared with a paired t-test. Statistical significance was defined with a two-sided *p*-value of < 0.05. Interobserver reliability was calculated with an intra-class correlation test for absolute agreement (results shown in supplemental Table 1). GraphPad Prism version 10 (GraphPad Software, La Jolla California USA), and IBM SPSS Statistics 26 (IBM, Armonk, NY, USA) were used for statistical analysis.

## Results

### Characteristics

Of the 45 healthy participants, handgrip data was available in *n* = 42, as two participants did not maintain consistent force during the exercise (supplemental Fig. 1), and one participant was excluded due to abnormal cardiac findings in the rest images. The age of the participants ranged from 19-43years, *n* = 20 (48%) were female, and mean BMI was 22.8 ± 2.9 kg/m^2^. The average sustained pressure during the handgrip was 14.7 ± 5.2 kPa.

### Haemodynamics and aortic function

Under the handgrip exercise, systolic (108±10mmHg to 121±13mmHg, *p* < 0.01) and diastolic (63±9mmHg to 78±13mmHg, *p* < 0.01) blood pressure significantly increased. Additionally, there was a small increase in heartrate (65±13 bpm to 67±10 bpm, *p* < 0.01). With the increase in blood pressure, both maximum and minimum aortic diameter increased during the handgrip exercise, reducing the aortic strain by a 16% relative change (31.0±11.3% to 26.1±12.3%, *p* < 0.01, Table 1). Distensibility dropped (6.4 ± 2.2 × 10^− 3^/mmHg to 5.4 ± 1.9 × 10^− 3^/mmHg, *p* < 0.01), and this led to an increase in the calculated vascular age from 36.5±9.9 years to 39.9±11.5 years (*p* < 0.01). Furthermore, a reduction in the maximum blood flow velocity and flow volume through the ascending aorta were observed (Table 1).

**Table 1:** Aortic Measurements

	Rest	Handgrip	%-Relative Change	*p*
Minimum aortic diameter (mm)	26.3 ± 4.6	28.0 ± 3.5	+ 6%	0.01*
Maximum aortic diameter (mm)	30.6 ± 3.3	31.4 ± 3.3	+ 3%	< 0.01*
Aortic strain (%)	31.0 ± 11.3	26.1 ± 12.3	-16%	< 0.01*
Aortic distensibility (x10^− 3^/mmHg)	6.4 ± 2.2	5.4 ± 1.9	-16%	< 0.01*
Vascular age (years)	36.5 ± 9.9	39.9 ± 11.5	9%	< 0.01*
Stroke volume (ml)	91 ± 19	86 ± 29	-	0.42
Maximum velocity (cm/s)	110 ± 22	102 ± 23	-7%	< 0.01*
Maximum flow (ml/s)	449 ± 117	440 ± 120	-2%	< 0.01*

Mean ± SD measurements from the ascending aorta. *p < 0.05 for comparisons between baseline and handgrip. The percent-relative change is shown for measurements with a significant change

### Ventricular and atrial function

**Fig. 3:**
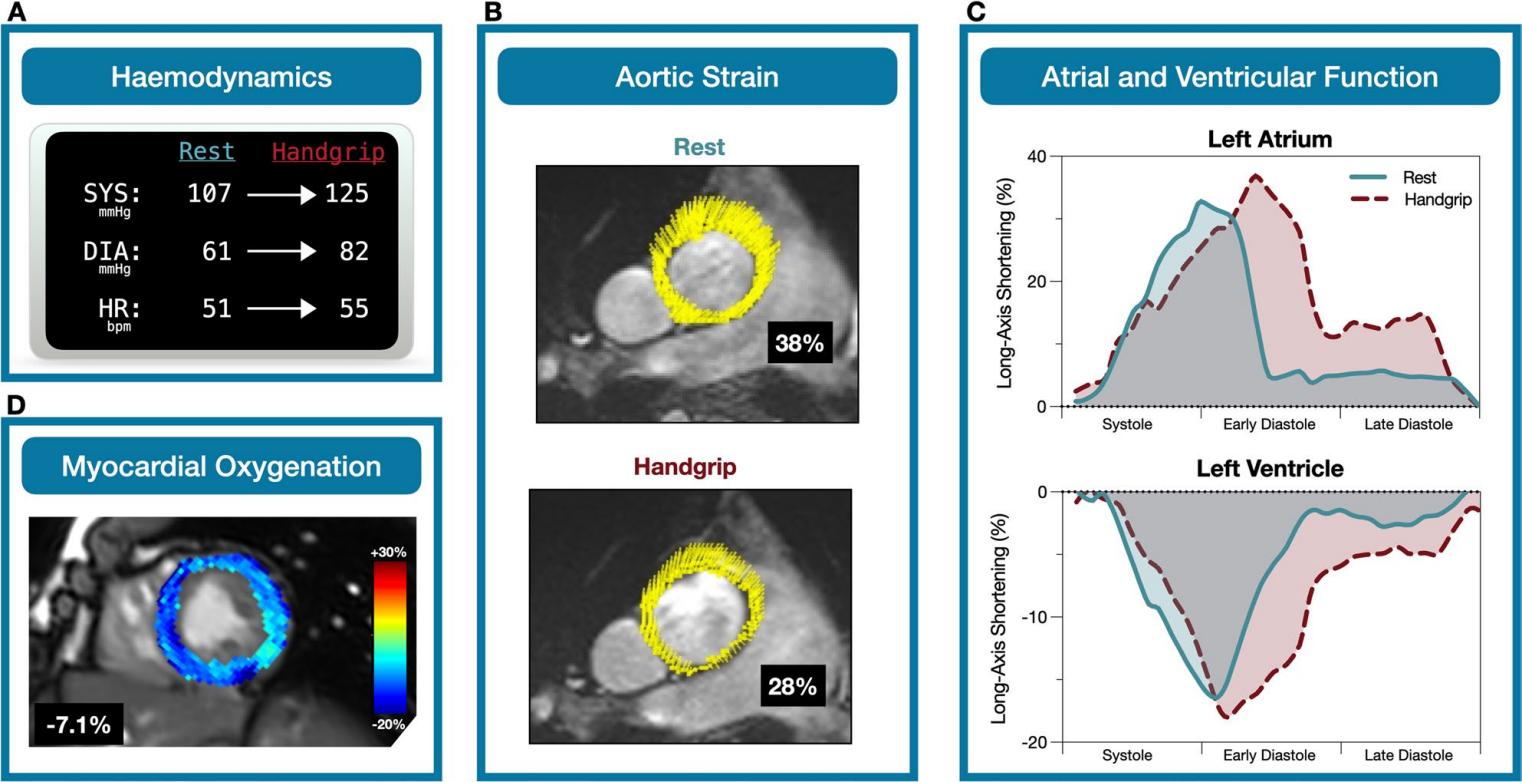
Patient example. **A**: Blood pressure and heart rate at rest (left) and during the handgrip (right). **B**: Aortic strain decreased from 38% to 28% under the handgrip. **C**: Left atrial (top) and ventricular (bottom) function increased during systole and late diastole with higher peak strain and greater atrial contraction. **D**. Myocardial oxygenation dropped 7.1% under the handgrip indicated by the blue overlay

From the standard volumetric analysis there was no change in biventricular ejection fraction (Table 2). Assessment of the chamber shortening (LAS and TAE_i_) measurements, which were indexed to chamber length, indicated an increase in LV (-18.4±2.6% to -19.3±3.3%, *p* = 0.04), and LA systolic function (36.2±6.4% to 39.6±7.9%, *p* < 0.01), resulting in a relative percent change of 5% and 4% from rest, respectively. For both chambers there was no change in early diastolic LAS, while late diastolic LAS corresponding to the atrial booster phase, increased in the LV by a 16% relative change (4.4 ± 1.7% to 5.1 ± 1.6%, *p* = 0.04), and by a 41% relative change in the LA (-11.5 ± 4.5% to -14.8 ± 5.3%, *p* < 0.01, Fig. 3).

**Table 2:** Ventricular and Atrial Function

	Left Heart				Right Heart			
	Rest	Handgrip	%-Relative Change	*p*	Rest	Handgrip	%-Relative Change	*p*
Ventricular Volumetry								
End diastolic volume index (ml/m^2^)	92 ± 17	91 ± 7	-	0.84	98 ± 21	99 ± 16	-	0.98
End systolic volume index (ml/m^2^)	41 ± 6	43 ± 5	-	0.09	56 ± 8	53 ± 5	-	0.60
Stroke volume index (ml/m^2^)	51 ± 13	48 ± 5	-	0.64	42 ± 18	46 ± 17	-	0.84
Ejection fraction (%)	55 ± 4	53 ± 4	-	0.34	41 ± 10	45 ± 11	-	0.72
Cardiac index (L/m^2^)	3.7 ± 1.1	3.9 ± 1.1	-	0.57	3.1 ± 1.6	3.8 ± 1.7	-	0.57
Ventricular Function								
Peak LAS/TAE_i_ (%)	-18.4±2.6	-19.3±3.3	-5%	0.04*	32.0 ± 4.7	33.5 ± 4.9	+ 5%	< 0.01*
Early Diastolic LAS/TAE_i_ (%)	14.5±3.1	14.4±3.7	-	0.76	-22.9 ± 5.0	-21.2 ± 4.7	+ 7%	< 0.01*
Late Diastolic LAS/TAE_i_ (%)	4.4±1.7	5.1±1.6	+ 16%	0.04*	-9.2 ± 2.4	-12.4 ± 2.7	-35%	< 0.01*
Atrial Function								
Peak/Reservoir LAS (%)	36.2±6.4	39.6±7.9	+ 9%	< 0.01*	44.1±10.6	46.8±7.7	+ 6%	0.02*
Early Diastolic/Conduit LAS (%)	-24.4±5.9	-24.7±5.8	-	0.98	-31.8±10.1	-31.6±8.0	-	0.75
Late Diastolic/Booster LAS (%)	-11.5±4.5	-14.8±5.3	-29%	< 0.01*	-12.2±5.2	-15.1±5.2	-24%	< 0.01*
Coupling Indices								
Ventriculo-Arterial Systolic Coupling	1.69 ± 0.65	1.39 ± 0.51	-18%	< 0.01*	-	-	-	-
Atrio-Ventricular Systolic Coupling	1.97 ± 0.33	2.07 ± 0.44	-	0.20	1.39 ± 0.28	1.41 ± 0.23	-	0.41

Left ventricular and biatrial function was reported using long axis shortening (LAS), while right ventricular function was reported by the tricuspid annular plane excursion indexed to right ventricular end-diastolic length (*TAE*_*i*_*).* Ventriculo-arterial coupling is a ratio of arterial stiffness (aortic strain, Table 1) divided by the absolute value left ventricular function (peak LAS), and a lower value indicates the left ventricle contracts more in comparison to the afterload. Atrio-ventricular coupling is calculated by the ratio of the peak atrial LAS divided by the peak ventricular LAS in the case of the left heart or TAE_i_ in the case of the right heart. The percent-relative change is shown for measurements with a significant change

*p < 0.05 for comparisons between baseline and handgrip

Similar findings were observed for the right side of the heart where there was a modest increase in peak systolic function by 5% relative change in the RV (RV-TAE_i_: 32.0 ± 4.7% to 33.5 ± 4.9%, *p* < 0.01) and 10% relative change in the RA (44.1 ± 10.6% to 46.8 ± 7.7%, *p* = 0.02). Additionally, late diastolic function increased with the handgrip by a 42% relative change in the RV (RV-TAE_i_: -9.2 ± 2.4% to -12.4 ± 2.7%, *p* < 0.01) and a 37% relative change in the RA (-12.2 ± 5.2% to -15.1 ± 5.2%, *p* < 0.01).

### Coupling indices

As a result of the handgrip test the ventricular-arterial systolic coupling index calculated between peak LV-LAS and aortic strain dropped (31.0 ± 11.3% to 26.1 ± 12.3%, *p* < 0.01), while there was no change in the atrio-ventricular coupling for either the left or the right side of the heart (1.97 ± 0.33% to 2.07 ± 0.44%, *p* = 0.20 and 1.39 ± 0.28% to 1.41 ± 0.23%, *p* = 0.41).

### Tissue characterisation

During the haemodynamic stimulus T1 and T2 relaxation times did not change (1193±25ms to 1194±22ms, *p* = 0.77 and 38.9±1.6ms to 38.7±1.9ms, *p* = 0.55 respectively, supplemental Fig. 2). However, tissue oxygenation slightly decreased by -2.9% [95%CI: -4.4, -1.3%, *p* < 0.01].

## Discussion

A five-minute handgrip exercise significantly increased blood pressure, resulting in measurable changes in myocardial oxygenation as well as aortic, atrial and ventricular function in healthy controls, quantified by CMR imaging. Late diastolic function, corresponding to the atrial kick phase of the cardiac cycle, was particularly enhanced during the handgrip exercise.

### Ventricular-arterial coupling

The primary effect of the isometric handgrip test on the ascending aorta was a transient increase in afterload due to elevated blood pressure, manifested as reduced aortic compliance, as indicated by decreased aortic strain and distensibility. This temporary increase in afterload was associated with a rise of several years in the derived theoretical vascular age, reflecting arterial stiffening comparable to that observed with physiological aging. This is expected in healthy cardiovascular systems during the stimulus. For example, reduced aortic elasticity has been observed as the physiological response to supine bicycle exercise in young healthy men [21]. In our imaging study, healthy hearts successfully compensated for the increased aortic resistance induced by the handgrip, as evidenced by an increase in peak LV-LAS while maintaining the same stroke volume as at rest. Previous echocardiographic studies using maintained and dynamic handgrip protocols also report increases in ventricular function [13, 22]. However, the simultaneous impact of the handgrip test on the ventricles and aorta has not been described until now. With the use of comprehensive CMR imaging, we were able to quantify ventricular-arterial coupling.

In healthy controls, this decoupling with ventricular function increasing more than the aorta during exercise, appeared to be beneficial, allowing the ventricle to adapt effectively. In contrast to the response of healthy adults in an awake setting, patients with cardiovascular dysfunction may struggle to compensate for decreased aortic compliance. Perioperatively it is well known, that the change in aortic elasticity and ventricular function impact ventricular-arterial coupling, which is a determinant of cardiovascular efficiency [23]. Afterload can be increased through surgical interventions like aortic cross-clamping [24], vasopressors such as phenylephedrine [25] or the patient’s stress response [26]. Moreover, ventricular-arterial decoupling in the other direction, due to worsening ventricular function, is associated with poor postoperative outcomes [27], thereby emphasizing the importance of assessing both left ventricular function and its interaction with systemic circulation. Moreover, this analysis focuses on functional parameters like longitudinal shortening and related strain measurements, which have been proven as superior to volumetric measures in perioperative and cardiac risk stratification [28–30].

### Atrio-ventricular coupling and diastolic function

While left ventricular function is routinely assessed in preoperative risk stratification, right ventricular dysfunction [31] and atrial dysfunction [32] have gained relevance in recent years as they both are independently associated with adverse outcomes. Therefore, it is important to have knowledge of abnormal function and gain a global picture of cardiovascular health preoperatively to adjust perioperative management accordingly. In both the right and left sides of the heart, systolic function increased in response to the handgrip stimulus, paralleled by an increase in peak biatrial function. As a result, systolic atrio-ventricular coupling indices remained unchanged. This indicates an expected response to the haemodynamic challenge, as effective coupling between the cardiac chambers maintains efficient cardiac function. Although cardiac testing focuses on ventricular function, atrial adaptability should not be overlooked as atrial dysfunction puts patients at increased risk for developing new onset perioperative atrial fibrillation [33].

The assessment of atrial function has emerged as an early diagnostic measure in diastolic dysfunction before ventricular changes are apparent [17, 34]. In our healthy participants, late diastolic function corresponding to the atrial kick, increased by more than 35%, compared to a 5–10% increase in systolic function across chambers. Diastolic dysfunction frequently represents an early manifestation of cardiovascular disease, even in asymptomatic individuals, highlighting its utility as a sensitive marker of subclinical cardiac dysfunction [35]. In the context of anaesthesia, assessment of diastolic function provides additional value beyond conventional evaluation of systolic function and may necessitate distinct perioperative management strategies [36]. Moreover, patients with diastolic dysfunction have been associated with increased risk for perioperative outcomes [37, 38].

### Myocardial tissue changes

Tissue characterization showed no change in myocardial or intravascular water content with the handgrip challenge, as indicated by stable T1 and T2 values [39]. Lu et al. also demonstrated that myocardial blood volume did not increase during the handgrip [15]. This suggests that myocardial perfusion did not increase. These findings align with coronary autoregulation, which maintains consistent coronary blood flow at perfusion pressures between 60 and 120 mmHg [40]. Yet, we did observe a slight reduction in myocardial tissue oxygenation. This is likely due to the increased oxygen demand arising from required inotropy to compensate for the increased afterload, without a corresponding increase in blood volume and oxygen supply [41]. This supports the concept that myocardial oxygenation may decline under increased oxygen demand from the handgrip test, even in healthy tissue.

### Potential of the handgrip exercise as a diagnostic tool

This study demonstrates that the handgrip exercise induces functional changes in the healthy heart and aorta in response to elevated peripheral afterload. We establish the feasibility of integrating this protocol with comprehensive whole-heart imaging. This approach has the potential to be further developed and applied in future studies involving patient cohorts, with the goal of establishing a technique with higher relevance to anaesthesia.

Diagnostic imaging research groups are introducing the handgrip exercise test in diagnostic imaging to avoid pharmacological agents [13, 22]. Ochs et al. compared a dynamic handgrip exercise protocol to dobutamine, an inotropic agent, which increases blood pressure through increased inotropy, in patients with suspected coronary artery disease and found that handgrip exercise offered high diagnostic accuracy, reduced examination time, and avoided drug-related adverse effects [14]. This work and other publications have observed a high interobserver reliability of the imaging measurements [42, 43], (supplemental Table 1), but further validation of the reproducibility of these cardiovascular responses is warranted. Handgrip testing has been shown to be reproducible in terms of maximal grip strength and associated blood pressure responses, yet cardiovascular imaging studies have not yet established whether these responses are reproducible [44].

While anaesthesiologists are unlikely to currently see findings in diagnostic clinical tests using a handgrip exercise, there are groups recommending using the handgrip for detecting various cardiovascular disorders such as coronary artery disease and diastolic dysfunction [45, 46]. Hypertension and blood pressure variability are both common in the operating room and are independently associated with adverse outcomes [3, 4]. Therefore, it is relevant to assess the response to these haemodynamic changes induced by the handgrip provocation and to avoid tests designed to induce hypotension, as the onset of symptomatic or severe hypotension should be prevented in a diagnostic setting whenever possible. It remains to be seen what information the handgrip test will show in patients with various cardiovascular pathologies and how the response will relate to perioperative outcomes, in particular haemodynamic instability. Our results focus on the feasibility of the isometric handgrip exercise to induce increased cardiovascular function in healthy participants and therefore does not allow for any statements about its prognostic or diagnostic value. It should be noted that our handgrip protocol primarily altered cardiac function, with only minimal changes observed in the tissue. By contrast, tests like adenosine infusion or breathing maneuvers (e.g., hypercapnia) may provoke more substantial myocardial tissue changes [19, 20]. Therefore, combining stimuli may better mimic perioperative stress and uncover additional diagnostic information.

Although CMR offers a comprehensive view of cardiac physiology and the number of CMR exams is rapidly growing [8], not all sites will have access to this advanced imaging modality. The handgrip test, however, is easily adaptable to other modalities like echocardiography, which, despite lacking tissue characterization, can still measure functional changes effectively.

### Limitations and implications for further research

This study was conducted exclusively in a healthy population; thus, we cannot draw conclusions about diagnostic or prognostic utility in patients with cardiovascular disease, or any potential influence of athleticism. The aim here was to demonstrate that this technique induces changes in healthy hearts and is feasible, with the goal that such a test can now be evaluated in a setting with cardiovascular and pre-operative patients in subsequent studies. In patients it would be interesting to assess if the handgrip triggers pathological mechanical decoupling or myocardial ischaemia. Future studies should evaluate if these changes observed in a diagnostic setting do predict perioperative complications.

## Conclusion

In this feasibility study of healthy volunteers, we showed that a 5-minute isometric handgrip exercise, assessed with comprehensive CMR, elicited significant changes in aortic compliance, myocardial oxygenation, and both systolic and diastolic ventricular function. By comprehensively assessing the cardiac and vascular response to this isometric exercise, it can be observed how efficient the cardiovascular system maintains atrio-ventricular-arterial coupling efficiency in the presence of intentional haemodynamic perturbations. This approach now warrants future studies investigating its effect in patients with known or suspected cardiovascular disease and to see if it could provide insight into how these multiple facets of the cardiovascular system react in the presence of fluctuating haemodynamics occurring perioperatively.

## Supplementary Information

Supplementary Material 1.

## Data Availability

Data is available upon request for scientists wishing to use them for non-commercial purposes, without breaching participant confidentiality.

## Abbreviations

CMR: Cardiovascular Magnetic Resonance
EF: Ejection Fraction
LA: Left Atrium
LAS: Long Axis Shortening
LV: Left Ventricle
MRI: Magnetic Resonance Imaging
RA: Right Atrium
RV: Right Ventricle
RV: TAEi–Right Ventricular Tricuspid Annular Excursion Index
