# Supplementary material for: Investigating the potential of the isometric handgrip exercise as a test of cardiovascular reactivity to blood pressure fluctuations
Source: BMC Anesthesiol. 2026 May 1;26:369. doi: 10.1186/s12871-026-03880-2 (PMC13281423; doi:10.1186/s12871-026-03880-2)
Supplement: Supplementary file 1 — Supplementary Material 1. [file 12871_2026_3880_MOESM1_ESM.pdf]

SUPPLEMENTAL MATERIAL

Investigating the Potential of the Isometric Handgrip Exercise as a Test of Cardiovascular Reactivity to Blood Pressure Fluctuations

Table of Contents

SUPPLEMENTAL METHODS 1: IMAGING DETAILS 2

SUPPLEMENTAL FIGURE 1: PRESSURE CURVES INDICATING SUCCESSFUL AND FAILED MAINTENANCE OF THE HANDGRIP 3

SUPPLEMENTAL FIGURE 2: BLAND-ALTMAN OF T1 AND T2 RELAXATION TIMES BETWEEN REST AND HANDGRIP 4

SUPPLEMENTAL TABLE 1. INTEROBSERVER RELIABILITY 5

Supplemental Methods 1: Imaging Details

Imaging was performed on a 3 Tesla Siemens Magnetom Prisma (Siemens Healthineers).

Ventricular and Atrial Imaging: Standard cine sequences were acquired in short-axis and long-axis planes of the heart for volumetric assessment (typical parameters: retrospective-gated, 30 phases, TR/TE: 3.1ms/1.38ms, flip angle: 45°, bandwidth: 962Hz/Px, spatial resolution: 1.9x1.9x8.0mm^3). A standard ECG retrospective gated balanced steady state free procession cine sequence was used during an end-expiration breath-hold (approximately 4-7s dependent on heart rate). A realtime cine was used for the acquisition of a short-axis stack (temporal resolution: 48ms, TE: 1.24ms, flip angle: 55°, bandwidth: 1260Hz/Px, spatial resolution: 2.8x2.8x8.0mm^3, one image acquired per heartbeat)

Tissue Characterization: Oxygenation-sensitive CMR images were obtained with an ECG-triggered balanced steady-state free precession sequence (TR/TE; 3.4ms/1.70ms, temporal resolution: 40.7ms, flip angle: 35°, spatial resolution: 2.0x2.0x10.0mm, bandwidth: 1302Hz/Px). A 5(3)3-modified Look-Locker sequence was used for T1 mapping, (TE 1.12ms, flip angle 35°, spatial resolution: 1.4x1.4x8.0mm, bandwidth: 1085Hz/Px). T2 maps were generated after acquiring three single-shot gradient echo images (TE: 1.32ms, flip angle: 12°; spatial resolution: 1.9x1.9x8.0mm, bandwidth: 1184Hz/Px with T2 preparation times of 0ms, 30ms and 55ms)

Atrial: The ascending aorta was assessed with a retrospective cine (30 phases, TR/TE: 3.2ms/1.37ms, flip angle 35°:, spatial resolution: 1.1x1.1x6.0mm^3). 2D flow of the same plane, was assessed with a prospectively gated phase contrast velocity-encoded gradient-echo sequence (VENC: 150 cm/s, TR/TE: 4.6/2.5 ms, flip angle: 15°, spatial resolution: 1.3x1.3x8.0mm, and temporal resolution: 38 ms).

CMR Analysis: For each type of measurement, a reader trained in the specific analysis performed the primary blinded analysis: long-axis shortening (A.C.Z.), mapping (M.D.N.), aortic 2D flow (L.S.), and myocardial oxygenation (J.A.). Twelve participants were randomly selected and re-analysed by independent readers (C.D.U., L.G., M.D.N., and A.C.Z.). All image analysts underwent training in these newer analysis techniques, and the first 25 participants were reviewed by senior investigators with more than 15 years of experience (D.P.G., B.J., and K.F.). Specifically for long-axis shortening, each plane was reviewed by consensus between the analyst and a senior investigator to verify that there was no foreshortening.

Supplemental Figure 1: Pressure curves indicating successful and failed maintenance of the handgrip

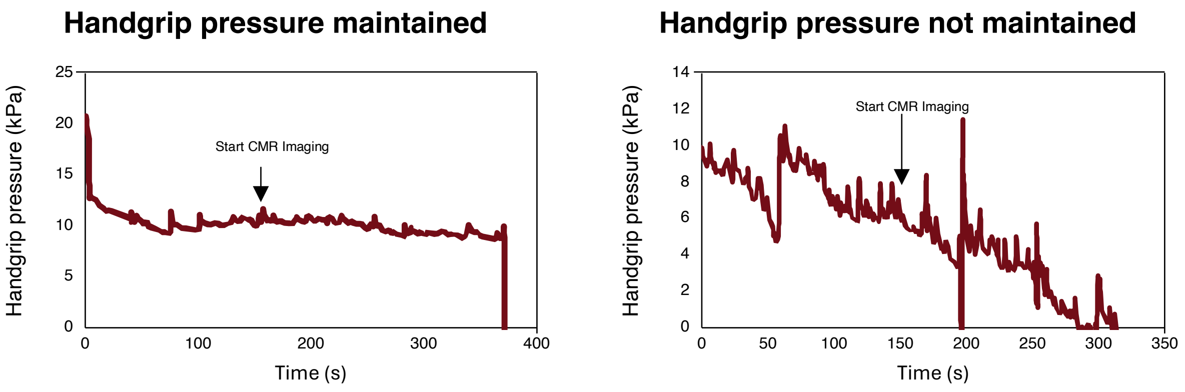

Left: Changes in pressure during the waiting period and the CMR exam in a participant, who was able to maintain a 30% of their maximal force throughout the exercise.
Right: Changes in pressure in a participant, who could not maintain the handgrip pressure and was therefore excluded.

Supplemental Figure 2: Bland-Altman of T1 and T2 relaxation times between rest and handgrip

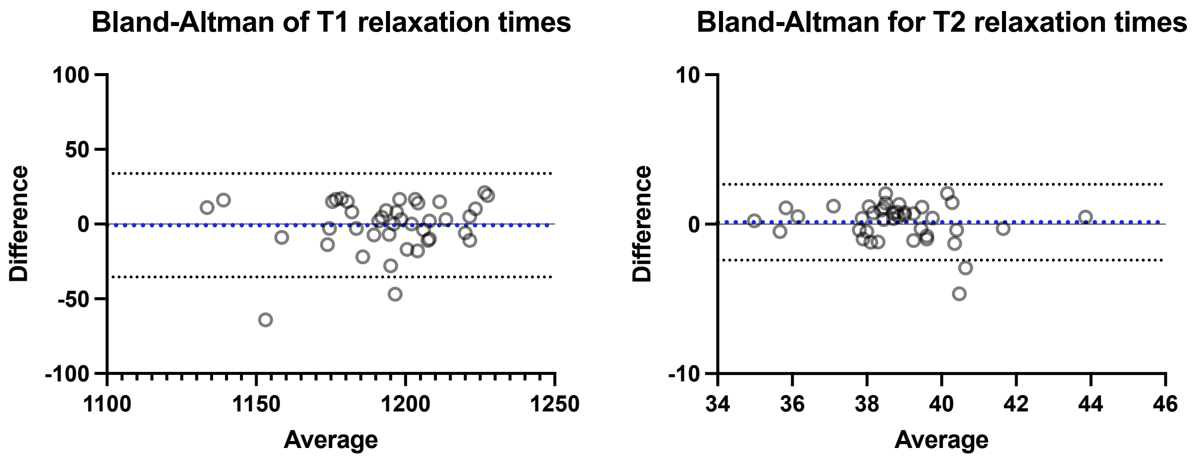

Table 2: The Bland-Altman plots for the T1 and T2 relaxation times (ms) are shown comparing measurements obtained between rest and the handgrip. The blue line represents bias with 95% agreement lines. The Bland–Altman plot showed an even distribution of differences around the zero line, with a mean bias close to zero, indicating no systematic difference between the two measurements obtained at rest and during the handgrip.

Supplemental Table 1. Interobserver reliability

A. Parameters assessed in the present study		
**Parameter**	**ICC**	**P value**
Myocardial oxygenation (%)	0.92	<0.01
T1 relaxation time (ms)	0.95	<0.01
T2 relaxation time (ms)	0.90	<0.01
Aortic stroke volume (ml)	0.99	<0.01
Aortic maximum flow (ml/s)	0.99	<0.01
Aortic maximum velocity (cm/s)	0.99	<0.01

B. Published values using the same technique		
**Parameter**	**ICC**	**P value**
**Ascending aortic measures (ARTFUN; Noda et al., 2016)**		
Maximum aortic area (cm^2)	0.96	<0.001
Minimum aortic area (cm^2)	0.95	<0.001
Aortic strain (%)	0.56	<0.01
**Long-axis shortening (Halfmann et al., 2024)**		
Left ventricular LAS (%)	0.97	Not reported
Left atrial peak LAS (%)	0.93	Not reported
Right atrial peak LAS (%)	0.89	Not reported

Intraclass correlation coefficients show excellent interobserver reliability was achieved for aortic and myocardial tissue parameters assessed from a sub-set of 12 participants presented in this study (top). Furthermore (bottom), publications using the same techniques and software as used in our study report also excellent inter-reader reliability for aortic, ventricular, and atrial measurements.
